# Compostable, fully biobased foams using PLA and micro cellulose for zero energy buildings

**DOI:** 10.1038/s41598-020-74478-y

**Published:** 2020-10-20

**Authors:** Kayode Oluwabunmi, Nandika Anne D’Souza, Weihuan Zhao, Tae-Youl Choi, Thomas Theyson

**Affiliations:** 1 Department of Mechanical and Energy Engineering, University of North Texas, Denton, TX 76207 USA; 2 Department of Materials Science and Engineering, University of North Texas, Denton, TX 76207 USA; 3 Tenstech, Inc, 424 Shrewsbury Lane, Matthews, NC 28105 USA

**Keywords:** Materials science, Biomaterials, Soft materials, Structural materials

## Abstract

Ecological, health and environmental concerns are driving the need for bio-resourced foams for the building industry. In this paper, we examine foams made from polylactic acid (PLA) and micro cellulose fibrils (MCF). To ensure no volatile organic compounds in the foam, supercritical CO_2_ (sc-CO_2_) physical foaming of melt mixed systems was conducted. Mechanical and thermal conductivity properties were determined and applied to a net zero energy model house. The results showed that MCF had a concentration dependent impact on the foams. First structurally, the presence of MCF led to an initial increase followed by a decrease of open porosity, higher bulk density, lower expansion ratios and cell size. Differential Scanning Calorimetry and Scanning Electron Microscopy revealed that MCF decreased the glass transition of PLA allowing for a decrease in cell wall thickness when MCF was added. The mechanical performance initially increased with MCF and then decreased. This trend was mimicked by thermal insulation which initially improved. Biodegradation tests showed that the presence of cellulose in PLA improved the compostability of the foams. A maximum comparative mineralization of 95% was obtained for the PLA foam with 3 wt.% MCF when expressed as a fractional percentage of the pure cellulose reference. Energy simulations run on a model house showed that relative to an insulation of polyurethane, the bio-resourced foams led to no more than a 12% increase in heating and cooling. The energy efficiency of the foams was best at low MCF fractions.

## Introduction

The carbon footprint of buildings is impacted by the material constituents and energy consumption. Material selection, manufacturing and thermal insulation performance all contribute to the carbon footprint. From a material selection perspective, a transition to renewable materials for material resources has driven increased interest in bioresourced options. In the case of insulation, petroleum-based polymer foam insulation such as polyurethane and expanded polystyrene are widely used due to their inherent properties^1–5^. To reduce carbon footprint, several bio-based polymers such as poly(caprolactone) (PCL), polyhydroxyalkanoates (PHAs), poly (butylene succinate) (PBS) and poly(lactide) (PLA) have been considered^6, 7^. PLA has had a significant commercial use because its raw material—lactic acid—can be produced by the fermentation of organic materials such as starch and sugars which are in abundance^6–8^. PLA requires about 25–55% less energy to produce than petroleum-based polymers^8^. Manufacturing carbon footprint has paired to quality of life concerns. Foam processing of thermoplastics is enabled through physical or chemical means. Physical blowing agents such as chlorofluorocarbons (CFCs), CO_2_ and N_2_ may be used to foam thermoplastics because they have a low boiling point, which provides sufficient vapor pressure for foam expansion at foam processing conditions. As CFCs are now known to be among the top five ozone-depleting substances, industries have turned to using inert CO_2_ and nitrogen. Chemical blowing agents are stable materials (usually solids) at normal storage temperatures but react to give off gas at their thermal decomposition temperature. Popular chemical blowing agents include azodicarbonamide, which produces nitrogen gas and small portions of carbon monoxide, carbon dioxide and ammonia. The gradual leaching of gases from chemical blowing agents into residential environments is an ongoing concern^9, 10^. The use of supercritical CO_2_ is growing as an option and its use is referenced as an environmnentally benign manufacturing process. Major applications of biobased foams are in biomedical and food packaging applications^6–8, 11, 12^. The processing parameters used in the production of porous composites from polymer via the supercritical CO_2_ (sc-CO_2_) foaming process have been reported to play significant roles in defining their morphology and mechanical properties^13, 14^. Using the extrusion foaming process with CO_2_ and N_2_ gas as a blowing agent, the mechanical properties of plain PLA foams were compared with those reinforced with joncryl chain extender. From the results, it was observed that the foams with chain extender inclusion showed increased elongation and overall improved tensile mechanical properties compared to foams without the chain extender^14^. Although PLA is a bio-based polymer with good processability it is slightly weak in toughness and thermal stability. Hence, to improve the mechanical properties of both solid and foam composites made from PLA, it has been blended with a range of natural fibers such as jute, kenaf, wood flour/fiber, cellulose crystals and whiskers^15–20^. For example, a comparison was carried out between the mechanical properties of treated epoxy bamboo fibers, vetiver grass fibers and coconut fibers and fibers that were used as reinforcement in PLA composites without being treated. The results obtained showed that an improvement in stiffness occurred in the untreated biocomposites compared to composites made with treated epoxy fibers^15^. Similarly, Huda et al.^20^ evaluated the physico-mechanical properties of PLA, reinforced with wood fiber composites produced through the micro-compounding molding system. The results obtained showed that the mechanical properties of the reinforced PLA composites were comparable to those of conventional polypropylene based thermoplastic composites with a maximum flexural modulus value of 8.9 GPa recorded at 30 wt.% fiber reinforcement. The use of cellulose as a reinforcement has been of significant interest due to its widespread availability from the paper and pulp industry^21–23^. This has been attributed to its natural abundance and ability to act as reinforcements at low filler concentrations^24–28^. Cellulose occurs in all plant-based materials as the principal structural component of the cell wall and can be chemically described as the linear polymer of (1–4)-linked *β-D-gluco-pyranosyl*residues^29–33^. The most common sources of cellulose are wood, agricultural crops and their residues such as wheat, straw, flax, hemp, sisal, soybean hulls, tunicates (animal cellulose), alga *valonia*, *oocystis, rhisophora apiculata* and *gluconacetobacter xylinus*^34–38^. By fibrillating cellulose pulp longitudinally, a three-dimensional network of cellulose popularly known as microfibrils can be produced^39, 40^. Cellulose microfibrils (MCF) have been produced using different techniques such as; micro fluidization, grinding, high-intensity ultra-sonication (HIUS), cryo-crushing, and steam explosion^41, 42^. MCF was first successfully produced by researchers in the 1980s. This was achieved by passing wood pulp suspension through a homogenizer several times to obtain highly fibrillated cellulose which was named micro cellulose^43, 44^. In order to increase, its hydrophobicity when used as a filler, MCF is often functionalized through chemical pretreatment with or without the use of enzymes^40^. Popular MCF functionalization methods include; acetylation and alkali-acid treatment^45–49^. Cellulose fibrils have sizes in the nanometer and micrometer range with diameters between 10 and 120 nm^40, 50–53^. Their large surface area (which is several hundred m^2^/g), high modulus of elasticity of about 150 GPa and high aspect ratio has stimulated their use as polymer reinforcements and fillers^54–56^. For example, Dri et al.^57^ used models based on the atomic structure to show that micro cellulose crystals have a stiffness of 206 GPa, which is comparable to that of steel. As a result of these properties, considerable research has been done in developing micro cellulose-based foams mostly for use in packaging applications^28, 58, 59^; and in medical research^60^. Many other examples have been documented to show how reinforcement of polymers with natural fillers have led to a corresponding improvement in mechanical properties^61–65^. However, for lightweight foam structures which are composed of a gaseous phase dispersed in a solid phase, achieving concurrent improvement in mechanical performance alongside other desirable insulation properties when natural fillers are added may be quite complicated^66^. This is because the overall properties of the foams which are dependent on the individual properties of both constituent phases are directly controlled by several factors. These factors include fiber/filler orientation, method of dispersion of filler in the polymer matrix, voids and porosity, pore structure, adhesion, interfacial interactions between filler and polymer matrix, and the percentage weight concentration of filler used^66–70^. These factors play major roles in determining the performance of foams when used for insulation and other applications. Cellulose possesses enormous potential for improving energy efficiency of buildings and reducing their environmental impact when used as insulation materials compared to petroleum-based insulation foams^71–75^. As a result, there has been renewed interest in developing sustainable building materials based on cellulose and other bioresourced polymers in recent times^76, 77^. The role of cellulose particles and fillers in supercritical CO_2_ foaming of PLA has been increasingly investigated most especially for foams made via the batch process^28, 78, 79^. In their work, Dlouha et al.^78^ suggested that there seemed to be an optimal density value for achieving high flexibility in PLA composite foams. To demonstrate this, they compared the tensile strength and bulk densities of composite foams made from amorphous PLA mixed with plain cellulose fibers and acetylated cellulose fillers at varying concentrations. The foams were made using CO_2_ gas as blowing agent for 6 h at a temperature and pressure range of 60 °C and (12–20) MPa respectively in the batch process. Results showed that while the foams made with acetylated cellulose gave higher bulk density with improved interfacial properties and tensile strength for up to 9 wt.% filler loading, the foams made with plain unfunctionalized cellulose fibers had a morphology with smaller average cell sizes and exhibited higher flexibility and toughness. In a separate work, they used nucleation theory to develop a linear relationship between the logarithm of cell density and the square of the foaming parameter to show that foams with higher bulk densities could be made at pressure values greater than 14 MPa^28^. Similarly, Ding et al.^79^ examined the rheology, thermal properties and foaming behavior of amorphous PLA/cellulose nanofiber (CNF) composites made through the solvent casting and CO_2_ batch foaming process with saturation temperature and pressure of 23 °C and 54 MPa respectively for 24 h. Their results showed that the ability for CNF to suppress cell coalescence produced a more uniform cellular morphology with smaller cell sizes and higher cell density compared to the pure PLA foam. However, beyond 3 wt.% CNF concentration, increase in stiffness which limited the foaming of the polymers was observed. Hussain and Dickson^80^ recorded about 3.5 times the original stiffness value due to strain hardening effects when semicrystalline PLA was reinforced with microcrystalline cellulose (MCC) at about 10 wt.% loading and foamed with liquid CO_2_ in the batch process. Uniaxially stretching the foams at different ratios showed that MCC was randomly oriented and well dispe rsed within the PLA matrix. Furthermore, other foaming techniques such as the freeze-drying has been used to evaluate the influence of cellulose on the morphology, mechanical and insulation properties of foams made from PLA and other polymers^77, 81, 82^. Kanno and Uyama^81^ demonstrated the effect of fiber orientation and extent of dispersion of the fibers on the mechanical properties of polylactic acid and bacterial cellulose (BC) cryogel monolithic foam composites made through the thermally induced phase separation (TIPS) and freeze-drying techniques. By controlling the porosity, monoliths having an ivy-like double network morphology consisting of entangled BC- fiber and PLA units were obtained. Higher compressive strength due to the presence of discontinuous pores and anisotropic 3D BC networks in the composite monoliths was also observed. Liu et al.^82^ summarized the dynamic effect of bulk density, cell morphology and interfacial properties on the deformational behavior of amorphous poly(vinyl) alcohol foams reinforced with cellulose nanofibers that were made using the freeze-drying method. Although, maximum compression strength was obtained at 30 wt.% cellulose nanofiber concentration, a consistent drop in bulk density, and a corresponding increase in percentage porosity was also observed in the foams. Yildrim et al.^77^, reported a thermal conductivity value of 0.045 W/mK and corresponding R-value of 3.14 for cellulose reinforced starch-based foams made using the freeze-drying technique. The ability for cellulose to act as nucleating agents when used as fillers as described in these various research works is also dependent on other factors such as their concentration, interactions with the matrix and foaming parameters such as temperature, pressure and time^68–70, 83^. In this paper we examine the effect of using MCF in PLA foamed using supercritical CO_2_ as a compostable building insulation panel. PLA is foamed using supercritical CO_2_ environmentally benign processing technique^40^. We use MCF in its unfunctionalized as-is state with no further processing to get a baseline impact of the microcellulose unaffected by additional chemically induced interactions. The mechanical and thermal properties were determined in the unfoamed and foamed materials. The energy consumption in a built environment was evaluated using Energy Plus applied to a model net zero energy house. The compostability was determined in accordance to international composting standards when landfilled at the end of their service lives.

## Materials and methods

### Materials

Amorphous PLA extrusion grade (Ingeo 4060D) with 12% d-lactide content (density 1.24 g/cm^3^ and melting temperature of 210 °C) was supplied by Nature works LLC, Minnetonka, MN. Micro cellulose fibrils were supplied by TENSTECH Inc. NC. They were sourced from Buckeye VFC SR-2711 acetate grade pressed, refined and bleached wood pulp at ~ 400 degree of polymerization. They were grounded to produce fibrils with size ranges of 10–120 nm and aspect ratio of 10. The effective MCF powder density was 0.47 g/cm^3^ as determined by the Hausen index method^84^. Carbon dioxide gas bone-dry grade (99.9% purity) supplied by Airgas was used as blowing agent. PLA/MCF blends containing MCF in weight fractions of 1.5, 2.25 and 3 wt.% were made. The nomenclature used for the samples is PLA_X with X = 0 (0 wt.% of MCF, 0 vol %), A (1.5 wt.% of MCF, 3 vol %), B (2.25 wt.% of MCF, 4.6 vol %) and C (3 wt.% of MCF, 6.1 vol %). The “f” in PLA_ Xf represents the foamed version of the composites. For biodegradation, the compost medium made of food waste and yard trimming was purchased from Denton County Municipal Waste Facility Texas.

### Foaming process

**Figure 1:**
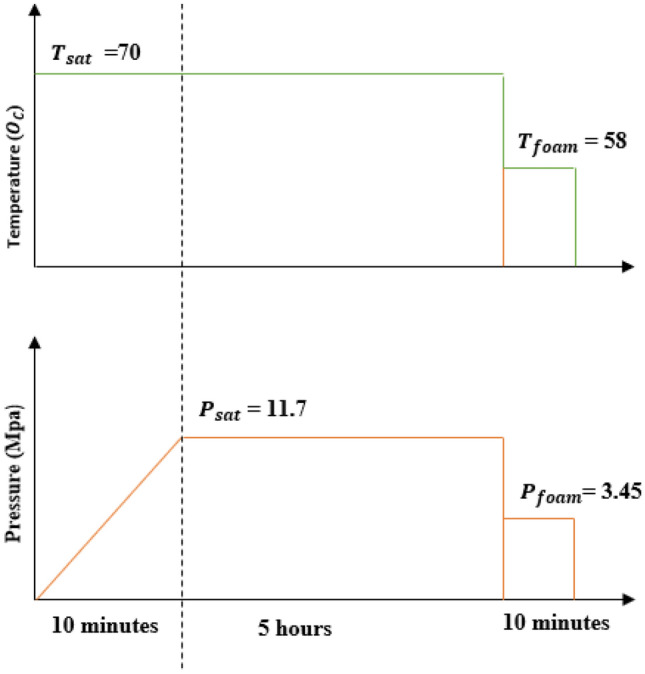
Schematics of the foaming process.

In order to foam the polymer blends, compression molded samples with diameter of 12.7 mm and thickness of 1.5 mm were made by melt compounding varying fractions of micro cellulose fibrils with PLA pellets using the twins screw extruder. Twin-screw mixing extruder made by Brabender Technologies was set to a working temperature of 200 °C for one hour to ensure that constant temperature for mixing was obtained. PLA was then introduced into the blender and mixed for 5 min until a free-flowing melt was obtained. Subsequently, the micro cellulose fraction was added. The PLA/MCFs blend was left in the Brabender for another 7 min for effective compounding to take place, after which the blend was removed and cooled for 30 min before it was crushed into smaller bits using a Fritsch pelletizer. This process was repeated for each of the fractions of MCF. Compression molded samples were subsequently made using the Carver hot press. Foaming experiments were performed using the two- stage CO_2_ pressure reduction process known as the decompression technique. The pressure vessel was pre-heated to a saturation temperature (T_sat_) of 70 °C with the samples in it for about 10 min for stabilization. CO_2_ was then released into the vessel at the saturation temperature for the soaking of the samples. A saturation temperature of 70 °C and saturation pressure (P_sat_) of 11.72 MPa were kept for a period of 5 h to ensure complete dissolution of the gas in the polymer. These values were chosen based on our preliminary experiments. At the end of the saturation time, the temperature was reduced drastically to foaming temperature (T_foam_) of 58 °C. At this temperature, first stage depressurization was initiated by quenching the pressure rapidly to foaming pressure (P_foam_) of 3.45 MPa, thus providing a driving force for cell nucleation and cell growth. Cell growth was promoted by leaving the samples in the vessel at the foaming temperature for 10 min. A second stage decompression was then carried out and the pressure vessel was simultaneously cooled to ambient temperature. The foams were subsequently removed from the vessel and prepared for characterization. Figure 1 below shows the schematics of the process involved in the production of the foams.

### Differential scanning calorimetry (DSC)

A Perkin Elmer DSC 4000 differential scanning calorimeter equipped with a Perkin-Elmer chiller was used for DSC measurements. Heating scans on the samples (weighing 8–12 mg) were performed at the rate of 10 °C per minute from 30 to 190 °C and held at 190 °C for 2 min. Then it was cooled down from 190 °C to 30 °C at the same rate. Two heating and cooling cycles were performed on each sample. Glass transition temperature (T_g_) was calculated from the heating scans^23^.

### Scanning electron microscope (SEM)

The morphology of the cross-section of prepared samples was examined in an environmental SEM (FEI Quanta 200 ESEM) under high vacuum conditions. To achieve this, the samples were initially freeze-fractured in nitrogen and the brittle edges were coated with 1.5 nm film of Gold/Palladium to make them conductive to obtain a clear image of the cross section of the PLA/CO_2_ composites. An accelerating voltage of 5 kV and working distance of 10 mm was used^38^. However, the foam skin thickness measurements were carried out in low vacuum conditions. This was achieved by preparing freeze-fractured sections of each specimen showing a clear-cut distinction between the skin and the core. Coating was not applied to the samples so that the skin edges will be clearly distinguishable.

### Foam density, porosity and morphological analysis

The unfoamed polymer and foam densities (*ρ*_p_ and *ρ*_f_) were determined using ASTM (D1505-98) and (D1622-98) standards, respectively^85–87^. Dahometer, electronic densimeter DH600 that is based on Archimedes principle of water displacement, was used to achieve this. Very insignificant water absorption was observed during the process due to the hydrophobic nature of the foam skin^88^. The relative foam expansion ratio was calculated as the ratio of densities of unfoamed polymer to that of the foams^89^.

1 $${\text{Expansion ratio}}\;{\text{Er}}\; = \frac{{\rho_{{\text{p}}} }}{{\rho_{{\text{f}}} }}$$

Percentage open porosity for each of the foams and cell type was obtained using the Ultrapyc 1200e model pycnometer made by Quantachrome Instruments Inc. It works on the principle of displacement of porous media filled with helium gas in the pycnometer according to Boyle’s law ^89, 90^.

#### Morphological analysis

The number of cells (bubbles) in each of the micrographs were counted using the Image J Pro software. For greater accuracy, each micrograph was divided into 4 sections. About 50 cells were counted randomly in each of the sections. Area (A) was then calculated for the total number of cells counted (n) in each micrograph. From the data obtained, the cell density (N_f_) for each of the foams was calculated using Eq. (2) below^79, 90^.

2 $${\text{N}}_{{\text{f}}} = \left( {\frac{{{\text{nM}}^{2} }}{{\text{A}}}} \right)^{3/2} \times {\text{Er}}$$

where A is in centimeter square; M is the magnification factor; and Er is expansion ratio.

The cell size for the foams (N_s_) was calculated from the Eq. (3) below^91^:

3 $${\text{N}}_{{\text{s}}} = \left( {\frac{{{\text{Er}} - 1}}{{{\text{N}}_{{\text{f}}} }}} \right)^{1/3}$$

Void fraction (V_f_) for the foams was calculated using the Eq. (4) below^92^:

4 $${\text{V}}_{{\text{f}}} = \left( {1 - \frac{1}{{{\text{Er}}}}} \right) \times 100$$

The cell wall thickness of the foams (C_wt_) was calculated using Eq. (5) below^93^:

5 $${\text{C}}_{{{\text{wt}}}} = {\text{N}}_{{\text{s}}} \times \left( {\frac{1}{{\sqrt {1 - \frac{{{\uprho }_{{\text{f}}} }}{{{\uprho }_{{\text{p}}} }}} }} - 1} \right)$$

### Mechanical testing (compression)

Measurement of compressive mechanical properties of the microcellular foams was performed on a Shimadzu AG-X plus series machine in compression mode at room temperature. The unfoamed plastic composites and foams were tested in accordance to ASTM D695 and ASTM D3574/D3575 respectively. Due to the brittle nature of the specimens, a crosshead speed of 0.5 mm/min was used to perform the compression tests. A lower value of 0.5 mm/min was taken after experimentation indicated that cracking ensued from using the 1.3 +/− 0.3 recommended by the standard. From the stress and strain curves, values of compression modulus and compression strength were determined^94^.

### Thermal conductivity measurement

To determine the thermal insulation performances of the foams, thermal conductivities were measured under room environment using a Hot-Disk thermal constants analyzer (TPS 1500) made by ThermTest Inc. The foam samples were cut into 50 mm diameter by 20 mm thickness dimensions. The samples were placed in such way that the Kapton sensor (with Ni spiral for heating), which was used as both the heat source (at 0.012 W power rating in the measurements) and temperature sensor, was sandwiched between two identical samples. The transient heat conduction test was carried out in an isotropic dual mode for 160 s. Thermal conductivity was calculated automatically by the instrument using the transient heat diffusion equation. To account for possible variation in heat flow rates across the foams based on the difference in morphology, tests were carried out from skin to core modes. Three different sets of each foam sample type were tested. To minimize deviation and ensure a high degree of accuracy in the results obtained, each experiment was repeated six times at 15 min interval for each sample making a total of eighteen tests per sample. The mean effective thermal conductivity (ƛ_*T*_) value and standard deviation of each sample set were then determined. The thermal resistance (i.e., R-value) was calculated as shown below^95^.6where T.R is the thermal resistance in m^2^K/W*;* t = thickness of specimen (m); and ƛ_*T*_ is the thermal conductivity of specimen (W/m∙K).

### Composting procedure

The biodegradability test of the foams was conducted according to ASTM D 5388-15 standard using the Automated Multi-Unit Composting System (AMUCS)^96^. To achieve this, compost soil was sieved and the + 250-micron oversize screen was selected for the experiment in order to obtain a homogenous sample. Moisture content, total and volatile solids analysis carried out on the compost in accordance to ASTM D 2974 standard was obtained as 56.5%, 45.51 (± 0.9) and 15.80 (± 0.8) respectively^97^. Using an Oakton Acorn pH 6 m the soil pH was calculated as 7.81 (± 0.3) and could be said to be slightly basic. 200 g of the compost representative fraction was weighted into each of the 500 ml Erlenmeyer flasks that was used as bioreactors and 2 g of pure PLA and cellulose reinforced PLA foams with dimensions of 1.5 cm × 1.5 cm were mixed with the compost in each flask. Pure PLA foam was used as the negative control, while 4 g of the analytical grade cellulose was also mixed with the compost in a separate flask and used as the positive control. Furthermore, three blank bioreactors comprising of compost soil alone were used as control samples. All samples were prepared in triplicates^96–98^. The contents of each bioreactor were thoroughly mixed with a spatula, weighted and incubated under optimal temperature and moisture content conditions of 57.2 ± 0.3ºC and 56.5%, respectively for 50 days. Regulated compressed air flow of about 0.2 standard liters per minute (slpm) was maintained throughout the experiment for adequate oxygen supply. Each bioreactor was weighted, and the water level replenished every 4 days to account for weight losses. CHN elemental analysis of the soil, foams and reference (pure cellulose) before and after the composting experiment was carried out by Atlantic Micro Labs Inc. (Norcross, GA) and the elemental composition for the compost was given as C = 16.18%, N = 0.64% and H = 4.84%; C = 17.07%, N = 1.63% and H = 3.27% before and after the experiment respectively. C/N for the compost was calculated as 25:1 and found to be within the recommended range of 10–40 ^96^. The results obtained from the CHN analysis were used in determining the percentage biodegradation of the foams using Eq. (7) below^98^.

7 $$\% \;{\text{ Biodegradation }} = \frac{{{\text{CO}}_{{2{\text{sample }} - {\text{ CO}}_{2} {\text{inoculum}}}} }}{{{\text{ThCO}}_{2} }}{ } \times { }100$$

where ${\text{CO}}_{2} {\text{ sample }}$ = accumulated amount of CO_2_ released from each bioreactor containing the samples ($\frac{{\text{g}}}{{{\text{vessel}}}}$), CO_2_ inoculum = accumulated amount of CO_2_ released from the control vessels ($\frac{{\text{g}}}{{{\text{vessel}}}}$), ${\text{ThCO}}_{2}$ = theoretical amount of CO_2_ calculated on the basis of total organic carbon content measurements.

## Results and discussion

### Effect of the inclusion of micro cellulose fibrils on glass transition temperature (T_g_)

As shown in Table 1 below, it was observed that an inclusion of varying weight fractions of MCF in the unfoamed composites resulted in steady decrease in their second T_g_ values when compared to that of pristine PLA. This was an evidence of plasticization of PLA by MCF in the blended unfoamed systems as seen in (PLA-A and PLA-B)^99, 100^. In comparison, as seen from the first DSC cycle of the foams, they were observed to have experienced further depression in T_g_ values due to plasticization by sc-CO_2_ leading to an increase in the mobility of the polymer chains^101–103^. All foams showed a decrease in T_g_ to be approximately 45 ± 2 indicating that two effects were operating. First the CO2 plasticized the PLA which showed a T_g_ depression in the pure PLA but the addition of MCF facilitated further permeation of CO_2_ resulting in a further drop in T_g_ of the PLA^28, 79^.

**Table 1:** The first T_g_ vales for the solid PLA/MCFs composites and second T_g_ values for the foams blends.

MCFs concentration (wt.%)	T_g2_	T_gf1_
0 (PLA-O)	60	54.4
1.5 (PLA-A)	56.09	47.09
2.25 (PLA-B)	52.23	45.89
3 (PLA-C)	45.45	45.66

### SEM microstructure of unfoamed and foamed pure PLA, PLA/MCF blends

**Figure 2:**
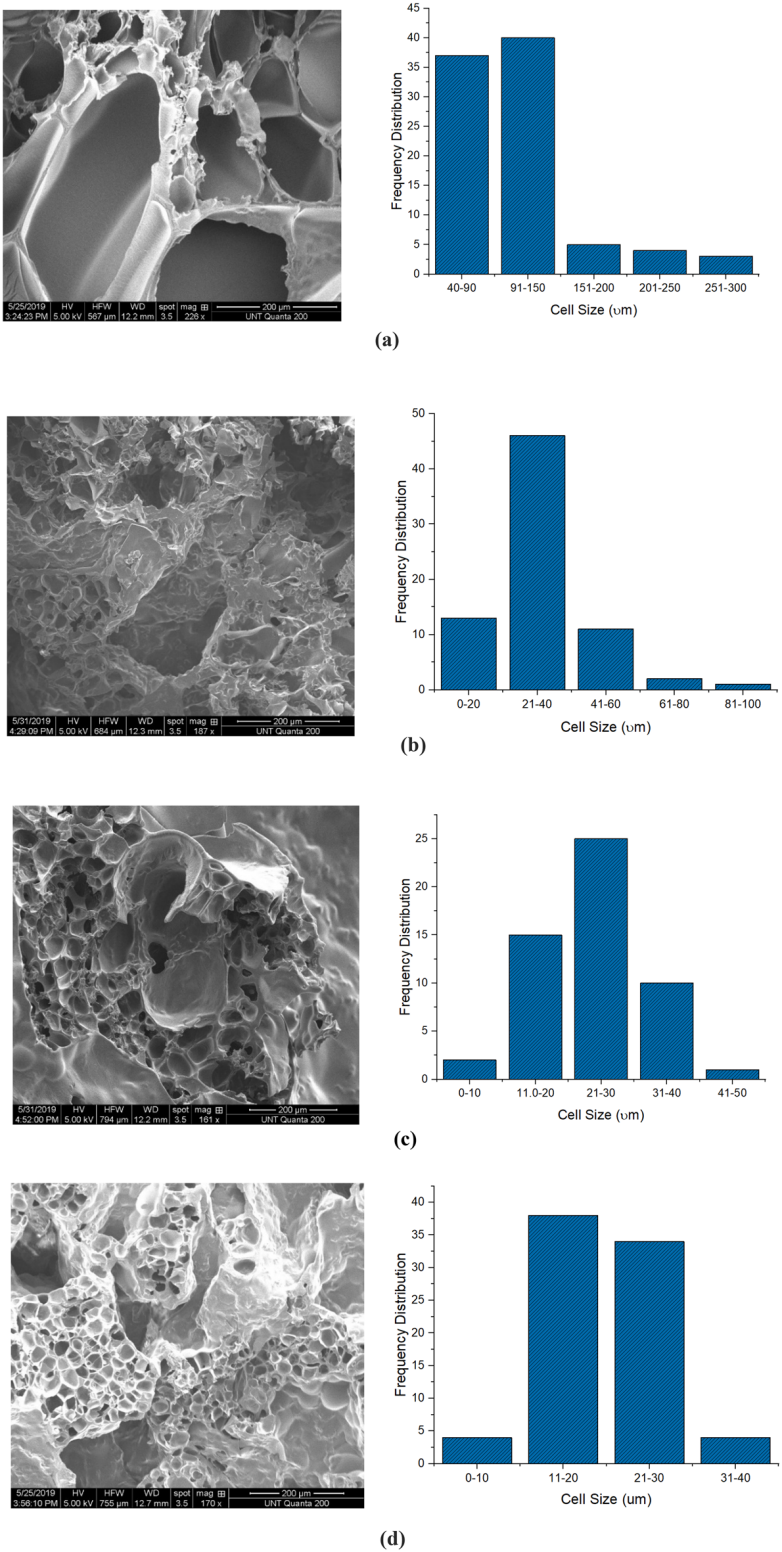
SEM micrograph and cell size vs frequency distribution: **(a)** PLA_0f., **(b)** PLA_Af, **(c)** PLA_Bf, **(d)** PLA_Cf.

Micro cellulose fibrils in the matrix of PLA acted as nucleating agents, due to the creation of nucleating sites and reduction in cell size^79^. This lowered the free energy required for bubble creation, causing increase in cell density of the foams as concentration of MCF increased in the matrix as displayed in Table 2 below^103^. It is also known that cell size is inversely proportional to the number of nucleation sites^104, 105^; this explains the increase in the number of cells per unit area with the corresponding reduction in cell size observed in the foams as the concentration of MCF inclusions increased as seen in Fig. 2 below^106, 107^. It was observed that the pure PLA foam had a good mix of large and small cell sizes. This was due to the two-stage decompression foaming technique that was used, which imparted a bimodal cellular structure which occurred through the simultaneous growth and resorption of bubbles^108^. Thermodynamically and as stated by the classical nucleation theory, bubbles with radius that are less than the critical radius require higher change in free energy (${\Delta G}$) to grow and so become re-dissolved in the polymer melt. However, with the introduction of a third phase in the form of MCF, ${\Delta G}$ was greatly reduced as reflected in the T_g_ values in Table 1 above^109^. This promoted heterogenous nucleation and reduction in foam cell size^110, 111^. Furthermore, the temperature gradient and shearing forces that characterized the processing route used in making the PLA/MCF composites foams which includes melt blending, pelletizing and compression molding and then foaming also contributed to the reduction in cell size, increase in cell density and variation in the bulk density gradient in the foams^112^. At 1.5 wt.% concentration, the micro cellulose fibrils were probably loosely held randomly at various interfaces in PLA leading to a reduction in the bulk density. The fibrils interacted with the liquid melt during the foaming process leading to over 200% reduction in cell size when foamed. However, as weight concentration of the MCF increased ‘hornification’ due to agglomeration at the nodes occurred^113^. This suggested that the rod-shaped fibrils started forming small pockets of 3-D networks, leading to a gradual increase in bulk density of the unfoamed composites (Table 2 below). This agglomeration could also be responsible for the decrease in void fraction and percentage open porosity that was observed in foams with higher weight percentage of MCF inclusions making the foams stiffer^113, 114^. This reduced the CO_2_ sorption and subsequent expansion in the foams^114^. A similar trend of results was documented for clay reinforced polypropylene foams^115^. As shown in the morphology table below it was observed that the foams were majorly open cell foams with percentage open porosity greater than 50% as obtained through the characterization of their percentage open cell content using the pycnometer^116^.

**Table 2:** Morphological parameters of the foams.

Foam type	PLA_0f.	PLA_Af	PLA_Bf	PLA_Cf
Average MASS of unfoamed polymer (g)	23.89 ± 0.01	16.36 ± 0.01	17.21 ± 0.01	17.45 ± 0.09
Average density of unfoamed polymer (g/cm^3^)	1.24 ± 0.01	1.21 ± 0.02	1.21 ± 0.02	1.23 ± 0.01
Average mass of foams (g)	15.17 ± 1.52	11.05 ± 0.82	13.57 ± 0.45	14.7 ± 0.7
Average density of foam (g/cm^3^)	0.2 ± 0.1	0.17 ± 0.03	0.27 ± 0.03	0.36 ± 0.02
Expansion ratio	6.33 ± 0.01	6.97 ± 0.01	4.5 ± 0.2	3.4 ± 0.1
Cell size (µm)	108.95 ± 13.24	34.19 ± 1.05	24.45 ± 2.31	20.89 ± 1.72
Cell density (cell/µm^3^)	4.45E−06	0.000106	0.000378	0.000451
Void fraction (vol. %)	84.2 ± 0.1	86.5 ± 0.2	77.8 ± 0.1	70.6 ± 0.1
Open porosity (vol. %) (pycnometer)	71.8 ± 0.1	76.13 ± 0.2	69.5 ± 0.1	60.28 ± 0.16
Cell wall thickness (µm)	9.73 ± 0.01	2.75 ± 0.02	3.29 ± 0.09	3.98 ± 0.08

### Effect of the inclusion of micro cellulose fibrils on mechanical and thermal properties

#### Mechanical properties

**Figure 3:**
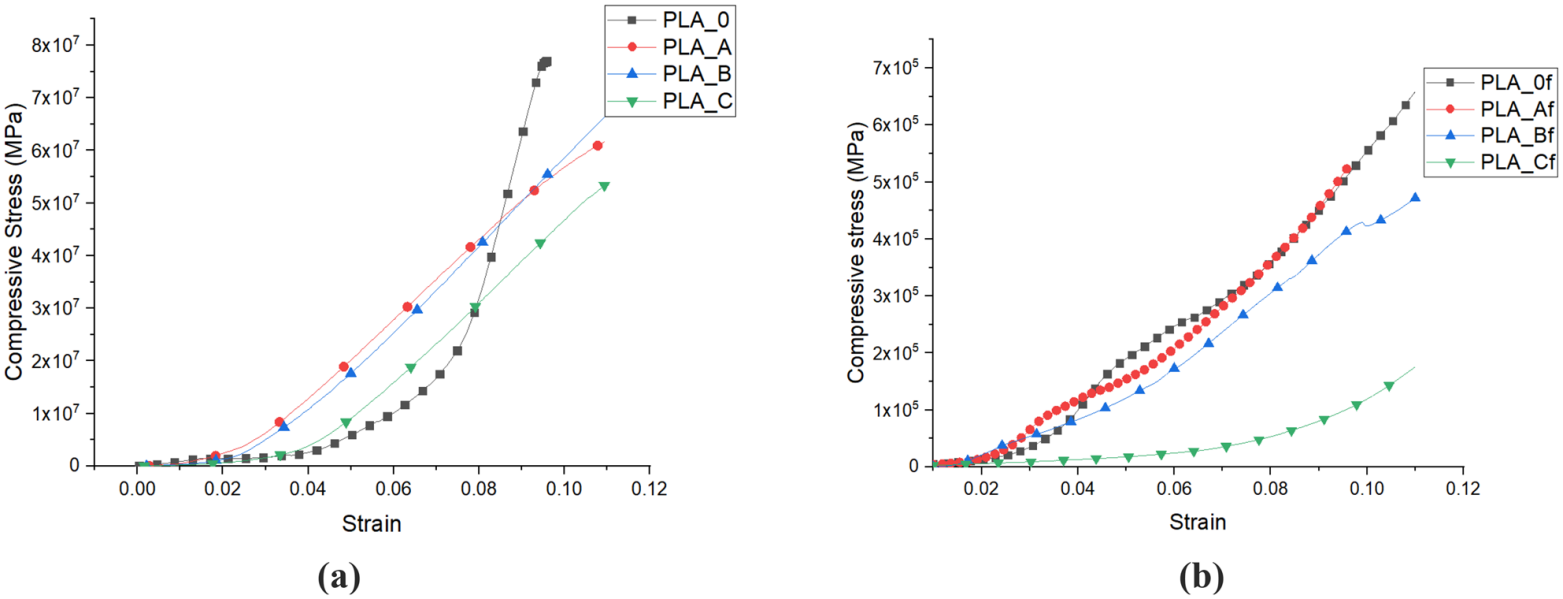
Plot of mechanical (compression) properties: **(a)** for unfoamed composites, **(b)** for foams.

Figure 3 and Table 3 show the results of the compressive tests of the unfoamed and foamed samples. In the unfoamed materials, the addition of MCF initially increases the modulus and strength by 48% and 35% respectively for PLA-A but further additions of MCF led to decrease in both to values below the original PLA. The corresponding foams showed a similar trend. Foams of PLA-A had a 20% and 19% increase in modulus and strength over the unfoamed materials. The results indicate that the foam mechanical performance was dominated by the material properties of the polymer. Pure PLA was improved through initial addition of MCF but higher MCF fractions led to diminished resistance to force in the compression testing mode. Interestingly all moduli and strength dropped ~ 99% when one compares the foamed to unfoamed materials regardless of MCF concentration. This indicates that the foam porosity changes from open to close cell or cell size was less contributive to the deformation of the foams. This deviates from the results observed in polypropylene foam where shorter and thinner cell walls with increasing cell size affected to foam mechanical performance^117^.

**Table 3:** Mechanical and thermal properties for the PLA/MCF composites with various weight fraction of MCFs.

Sample type	Compression modulus (MPa)	Compression strength (MPa)	Thermal conductivity (W/m∙K)	Cp (J/kg·K)	Thickness (m)	R-values (m^2^K/W)
PLA_0	100 ± 1.5	2 ± 0.2	0.191 ± 0.425	251	0.03 ± 0.01	0.65
PLA_A	148 ± 1.9	2.7 ± 0.4	0.162 ± 0.144	359	0.02 ± 0.01	0.62
PLA_B	80 ± 1.7	1.5 ± 0.2	0.179 ± 0.342	254	0.02 ± 0.01	0.56
PLA_C	60 ± 1.3	0.82 ± 0.03	0.183 ± 0.420	296	0.02 ± 0.01	0.55
PLA_0f.	0.83 ± 0.05	0.021 ± 0.001	0.058 ± 0.470		0.02 ± 0.01	1.73
PLA_Af	1 ± 0.1	0.025 ± 0.004	0.049 ± 0.091		0.02 ± 0.02	2.03
PLA_Bf	0.53 ± 0.04	0.018 ± 0.005	0.068 ± 0.511		0.02 ± 0.01	1.41
PLA_Cf	0.41 ± 0.1	0.014 ± 0.001	0.074 ± 0.252		0.02 ± 0.02	1.25

However, it was observed that an increase in weight fractions of MCF in the composites caused a reduction in compressive modulus and strength. The PLA-B experienced a 20% reduction in compressive modulus for the unfoamed composite and a corresponding 42% reduction in the foamed composite respectively compared to pure PLA composites. Similarly, the PLA-C fraction experienced 34% reduction in compressive modulus of the unfoamed composite as shown in Table 3 below. This translated into about 60% reduction in the compressive modulus of the foamed composite. This trend of results may also be due to a reduction in non-uniform dispersion of the natural fibrils in the polymer matrix and their micro-aggregation at the nodes as the weight fraction of the MCFs increased, leading to poor interfacial bonding and adhesion between the fibrils and PLA molecules^118, 119^.

#### Thermal properties

It was observed that the inclusion of micro cellulose fibrils in PLA influenced the density of the unfoamed composites leading to a linear increase in effective thermal conductivity as the weight concentration of the inclusions increased as shown in Table 3 below. The thermal conductivity of unfoamed PLA gave a value of 0.191 W/m∙K that falls in the (0.1–0.5) W/m∙K documented range for non-conducting polymers due to their amorphous and defective internal structure^120, 121^. The reduction in the thermal insulation property of the PLA-Af foam could be attributed to a drop in its density which is due to the poor interfacial interaction between the amorphous regions of the fibrils and PLA which reduced the Van der Waals forces between them^122^. The inability for MCF to form inter-filler networks with PLA at this concentration increased the interfacial thermal resistance^123^. However, as the concentration of MCF increased, the fibrils began to agglomerate^119^; this occurred at the nodes leading to higher mass and bulk density and a corresponding increase in effective thermal conductivity. A similar trend was also observed for the foams. The mean effective thermal conductivity (ƛ_*T*_) of foams could be represented by different mechanism taken separately as shown in Eq. (8) below^124^:8
where ƛ*sol*  = conduction through the solid phase, ƛ_g_ = conduction through the gas phase, ƛ_*r*_= thermal radiation and ƛ_cv_  = convection in the gas phase = 0 (since the cell size of the foams are < 4 mm)^124^. The 1.5 wt.% MCF foam also gave the lowest mean effective thermal conductivity value of about 0.04926 W/m∙K which was about 15% lower than that of the pure PLA foam as shown in Table 3 below. Beyond this, the mean effective thermal conductivity values of the foams increased as the concentration of MCFs in the PLA increased. The large cell sizes of the pure PLA foams led to some degree of radiation from the foams^125^. Nevertheless, the large volume of air in the cell greatly reduced the conduction heat transfer rate and was responsible for the low density of the foam, which contributed to its low thermal conductivity^126^. Density and void fractions were the major factors that determined the resultant mean effective thermal conductivity of the foams. In addition to the poor interfacial adhesion and the absence of networks between cellulose fibrils and PLA atoms^127, 128^, the hydrophobicity of the non-polar side chains of the CO_2_ foaming agent and non-polar amorphous nature of PLA made PLA to dissolve extensively in the foaming agent leading to greater foamability since ‘like dissolves like’^129, 130^. This resulted in bigger cell sizes and higher open porosity as recorded for pure PLA and 1.5 wt.% MCFs foam and resulted in lower thermal conductivity. MCF consists of both crystalline and amorphous regions. Although the weight fraction of MCF used in PLA was small, the amorphous regions of the MCF also contributed to the poor interfacial adhesion. For the 1.5 wt.% MCF foam, the fibrils were located at the interface and their perforation of the cell wall led to a higher percentage open porosity and void fraction. This increased the volume of air in the foam, lowered its density and made it more thermally insulative^131^. However, as the concentration increased, the MCF started aggregating at the nodes^129, 130^. Due to their rod-like shape, they were able to form small pockets of 3-D networks leading to a reduction in cell growth, cell size and void fractions. A subsequent increase in bulk foam density led to a corresponding increase in mean effective thermal conductivity for the 2.25 and 3 wt.% MCF foams (PLA-Bf and PLA-Cf). As it has been stated that the cell density, cell size, and size distribution contribute to the final bulk density of the foamed product^131^, and therefore, the effective thermal conductivity.

**Figure 4:**
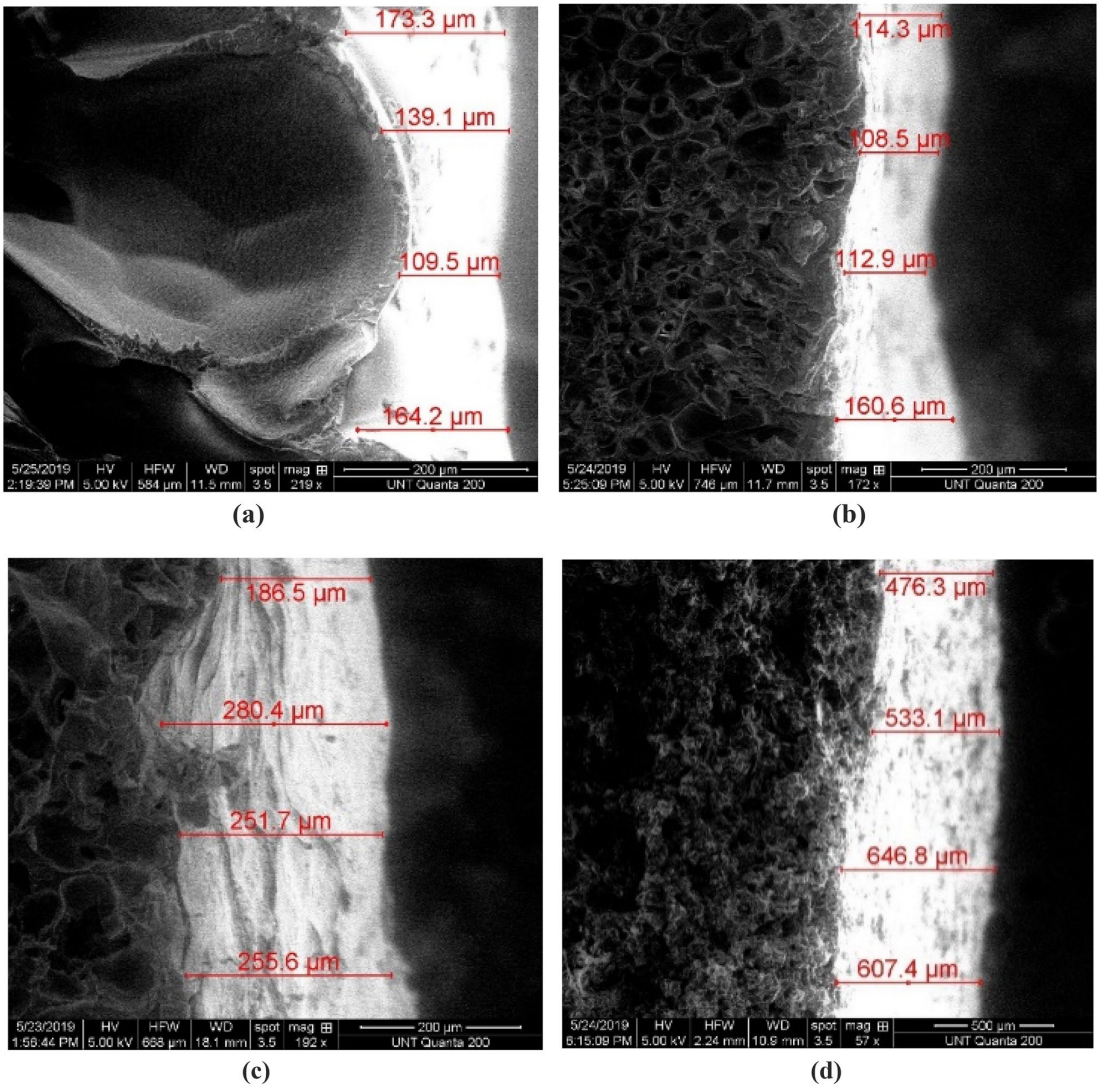
Skin thickness for **(a)** PLA_0F, **(b)** PLA_Af, **(c)** PLA_Bf, **(d)** PLA_Cf.

The aggregation of micro cellulose fibrils at the nodes and edges also increased the thickness of the foam skins and reduced the percentage porosity as shown in the skin thickness SEM images shown in Fig. 4 below^132, 133^. This further contributed to an increase in conduction in the solid polymer and contributed to the higher mean effective thermal conductivity documented for the foams with higher MCF fractions.

The results reflect that thermal conductivity first decreased below that of PLA when micro cellulose was first introduced but that higher micro cellulose content led an increase in thermal conductivity over PLA in the foamed materials. The higher thermal conductivity with higher micro cellulose was found to be related to the increase in skin thickness and thicker cell walls with higher micro cellulose content. The skin and cell wall fraction increased the effective polymer fraction in the foam leading to increased thermal conductivity. To validate this hypothesis, the effective thermal conductivity of the foam was determined using the unfoamed polymer and void fractions as contributors. A number of empirical and theoretical models have been developed for predicting the effective thermal conductivity of solid composites that are considered as two-phase mixtures or continuous phase systems^134–139^. Many of them have been used to efficiently predict the thermal conductivity values of various polymeric composites with fillers at low concentration^140–142^. The theoretical thermal conductivity (ƛ_*T*_) of the foams was computed using the correlation to occupied volume in Eq. (9)^143, 144^:9 where $\theta_{{\text{p}}}$ = Volume fraction of the polymer matrix; ƛ_p_ = thermal conductivity of the polymer matrix (W/m∙K); ƛ_air_ = thermal conductivity of air (W/m∙K) = 0.0267. The results (Table 4) showed the mean effective thermal conductivity values across each foamed sample. The theoretical results followed the same trend as the experimental measurements with the standard deviation being between 2 and 10% over that of the theoretically predicted from Eq. (8).

**Table 4:** Comparison between theoretical and experimental measured thermal conductivity values.

Sample type	Theoretical Values (W/m∙K)	Experimental Values (W/m∙K)	Difference (%)
PLA_0f.	0.0527	0.05774	9
PLA_Af	0.045	0.0493	9
PLA_Bf	0.0605	0.06755	10
PLA_Cf	0.0728	0.07408	2

**Figure 5:**
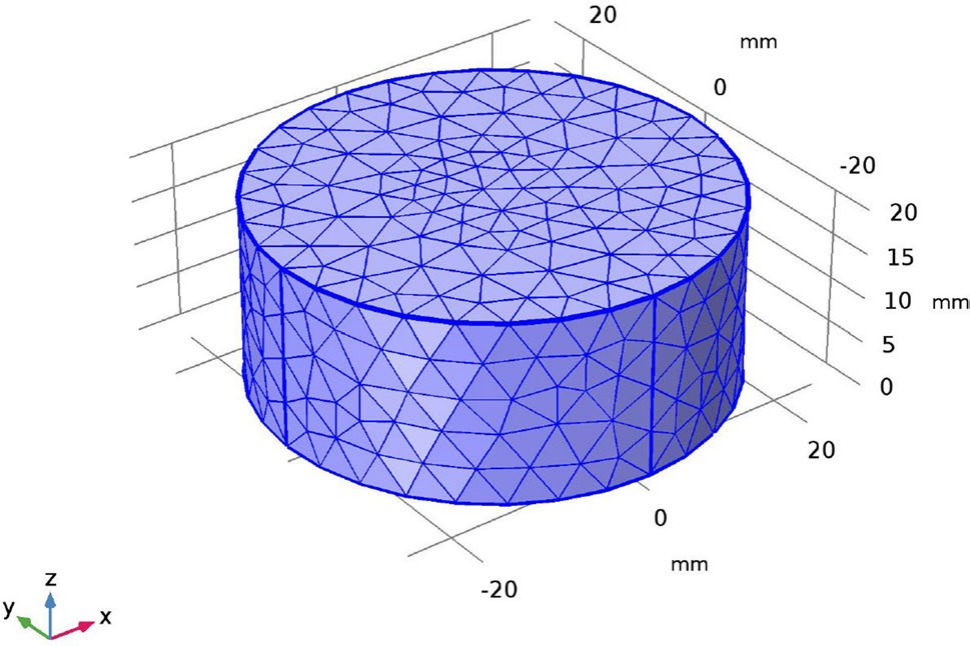
Schematic of the 3D cylindrical geometry model with the tetrahedral mesh structure used for simulations.

To estimate the impact of the foam on the insulation characteristics, simulations were carried out using predefined finite element functions for heat transfer analysis in porous media available in COMSOL Multiphysics software version 5.3a. The basic idea of the model was that each foam system was an isotropic continuous phase system which was made up of a porous polymeric material (a blend of PLA/MCF composite) filled with random pockets of air. The percentage of void fraction obtained from the morphological table (Table 2) indicates the total amount of air in each composite. A cylindrical geometry was used for each model since this was the shape of the experimental foams. The free tetrahedral mesh function in the software was used for the random pore distribution (shown in Fig. 5 below). Unfoamed values of the density, thermal conductivity and specific heat of the polymer paired to porosity were input into the simulation. The thickness and radius of the foams were taken as 20 mm and 25 mm, respectively, which were similar to the sample dimensions used for the measurements. For the boundary conditions, heating power of 0.012 W was applied at the top surface of the specimen and the other boundaries of the sample were held in natural convective heat losses to the environment.

**Figure 6:**
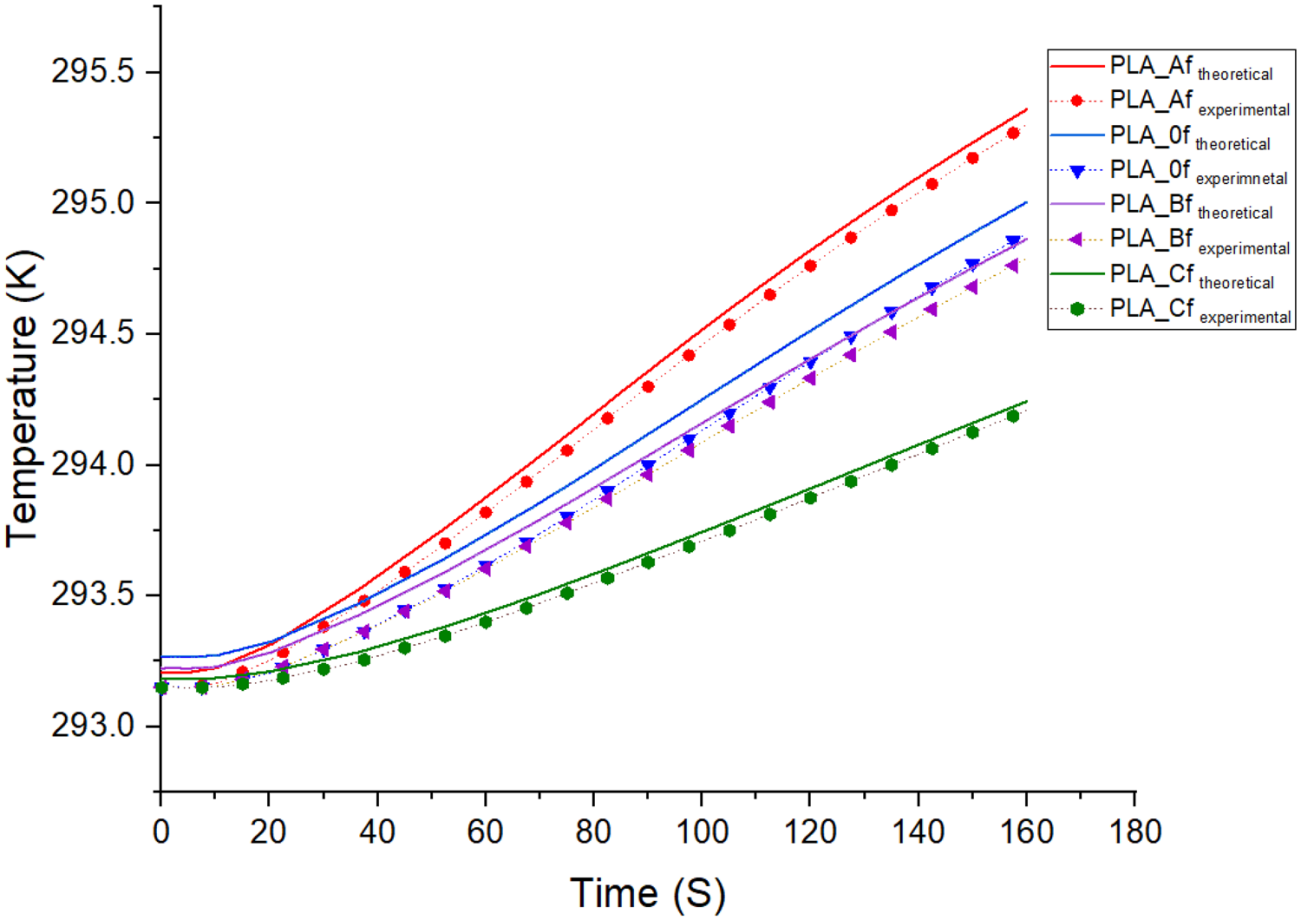
Plot of temperature vs time for both theoretical and experimental data input for the composite foams (at the middle location of foams).

Figure 6 below displays the temperature variations in various samples. The simulated temperature –time profile of the foam shows similar output from the temperature–time profile generated using experimental specific heat, density and thermal conductivity of foams. As seen from the slope of the samples, the temperature of the foam sample with lower thermal conductivity value (higher R-value) rises faster than that of foam sample with higher thermal conductivity value (lower R-value) during the heating process (0.012-W heating power input). This demonstrates that the high thermal resistance (R-value) material has lower heat dissipation rate to the surrounding to maintain the thermal energy. Therefore, high R-value insulation can help remain the indoor temperature with low heat loss/gain rate to/from the outer environment for building energy savings.

### Energy modeling for the micro cellulose fibrils reinforced PLA foams

**Figure 7:**
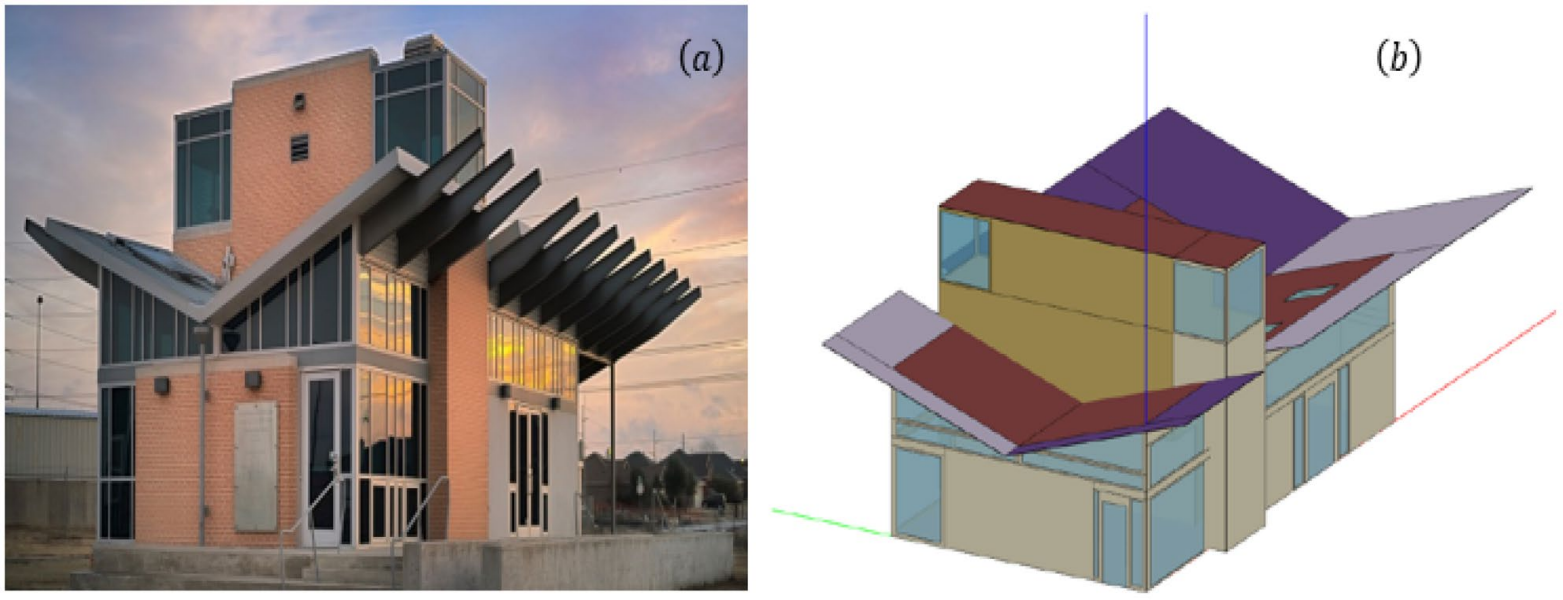
**(a)** UNT’s Zero-Energy Research laboratory; **(b)** ZØE building model in SketchUp for Energy Plus simulation. (Photograph 7a is used with permission. credit to UBSC/UNT).

To estimate the energy footprint impact of the foams on the built environment, the Net Zero Energy House simulated environment was used to determine the heating and cooling energy usage relative to conventional VOCs containing non-renewable, non-bio-resourced foams like expanded polystyrene and Polyurethane. The Zero-Energy (ZØE) Research Lab at University of North Texas shown in Fig. 7 below is a physical building environment, which has been modeled to study its energy consumption^145^. It was used as the baseline for the energy modeling in EnergyPlus. The new micro cellulose enhanced PLA foams were used to replace the insulation layers in the building envelope in the model in order to investigate their insulation performance for energy savings. The construction materials and energy consumption properties of the house are fully known. The ZØE House is a 111.48-m^2^ sustainable, energy-efficient house that provides researchers a variety of renewable energy sources, energy-efficient building materials, and state-of-the-art equipment/instrumentations, such as solar panels, solar water heaters, geothermal heat pumps, underfloor radiant heating and cooling, solar chimney, rainwater harvesting and water purifier system, etc. The ZØE lab provides a carefully controlled environment for the studies on human/building interactions and energy harvesting technologies in buildings, i.e., solar, wind, geothermal, etc. The ZØE building was designed in the exclusive architectural commercial design software SketchUp, stretching over a 111.48-m^2^ area with 3.66-m ceiling height space (Fig. 7). The model consisted of three simulation zones: conditioned zone, mechanical room, and electrical room. Two types of insulation exist in the ZØE lab wall structure (shown in Table 5). One is a 100-mm structural insulated panel (SIP); the other is a 150-mm batt insulation in the masonry wall. Both insulations are polyurethane foam-based material and have good thermal insulation performance with the R-value of about 5 per inch thickness. They are in the east, north and west facades of the building. EnergyPlus version 8.9.0, an industry recognised energy modeling software, with inbuilt insulation materials similar that of the ZØE and inbuilt ideal load permiting HVAC systems to act at 100% efficiency was used for the analysis. Constant temperature set points of 24 °C with two modultaing setpoints: 23/25 and 22/26 °C and compared to that of summer design day in Dallas-Forth worth, Texas was used for the simulation^145^.

**Table 5:** Wall construction layers in the ZØE lab at UNT.

	**SIP WAll**	**Masonry wall**
Layer 1	100-mm SIP	100-mm Brick
Layer 2	Air Gap	Air Gap
Layer 3	15.88-mm Thick Gypsum Board	12.7-mm Thick Sheathing
Layer 4		150-mm Batt Insulation
Layer 5		15.88-mm Thick Gypsum Board

The thermal properties of the new micro cellulose reinforced PLA foams used to replace the current two types of insulation in the building wall structure (the baseline) in the Energy Plus model to investigate energy consumptions of building protected by the proposed new foams, are shown in Table 6. SIP/insulation values reflecting conventional foams in the ZOE model home provide the reference framework. Comparative energy costs were investigated through replacement of the conventional SIP material with the foams.

**Table 6:** Thermal properties of various insulations.

	Density (kg/m^3^)	Specific heat (J/kg∙K)	Thermal conductivity (W/m∙K)
4″ SIP/6″ Batt insulation	16/24	1300/1210	0.033/0.0288
PLA_0f.	195	524	0.05774
PLA_Af	173	567	0.04926
PLA_Bf	270	412	0.06755
PLA_Cf	363	407	0.07408

**Figure 8:**
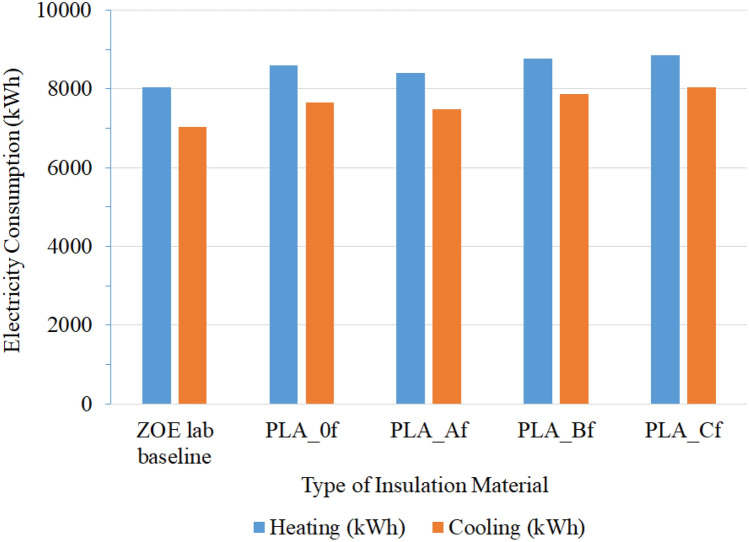
The annual heating and cooling energy consumptions in the ZØE lab using the new micro cellulose reinforced PLA foams as the insulation material in the wall construction.

The results obtained as shown in Fig. 8 below gave an estimate of the annual heating and cooling loads in the ZØE lab with different insulation foams embedded in the building envelope. The results of the simulations indicated that the proposed micro cellulose reinforced PLA foams provided similar building energy protection as the traditional polyurethane foams, with only about a maximum of 12% increase of energy consumption. However, the new foams can be beneficial to the environment compared to conventional insulation materials. Overtime, the inclusion of micro cellulose fibrils in the PLA foam could potentially lead to more energy savings in buildings compared to pure PLA foam. The foam with lower loading of micro cellulose was the best.

### Biodegradability of the foams

**Figure 9:**
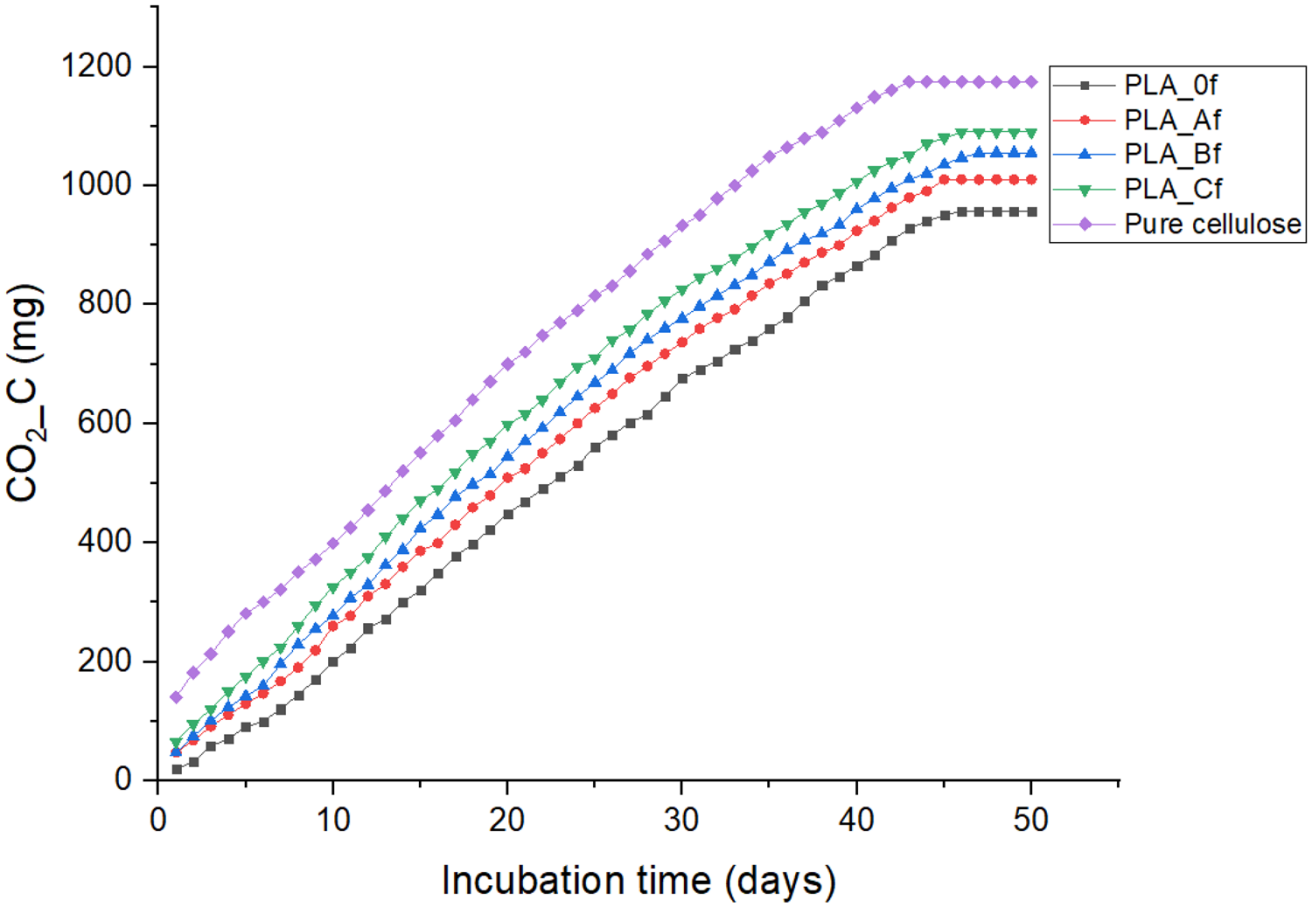
Net cumulative CO_2_-C production for the foams.

**Figure 10:**
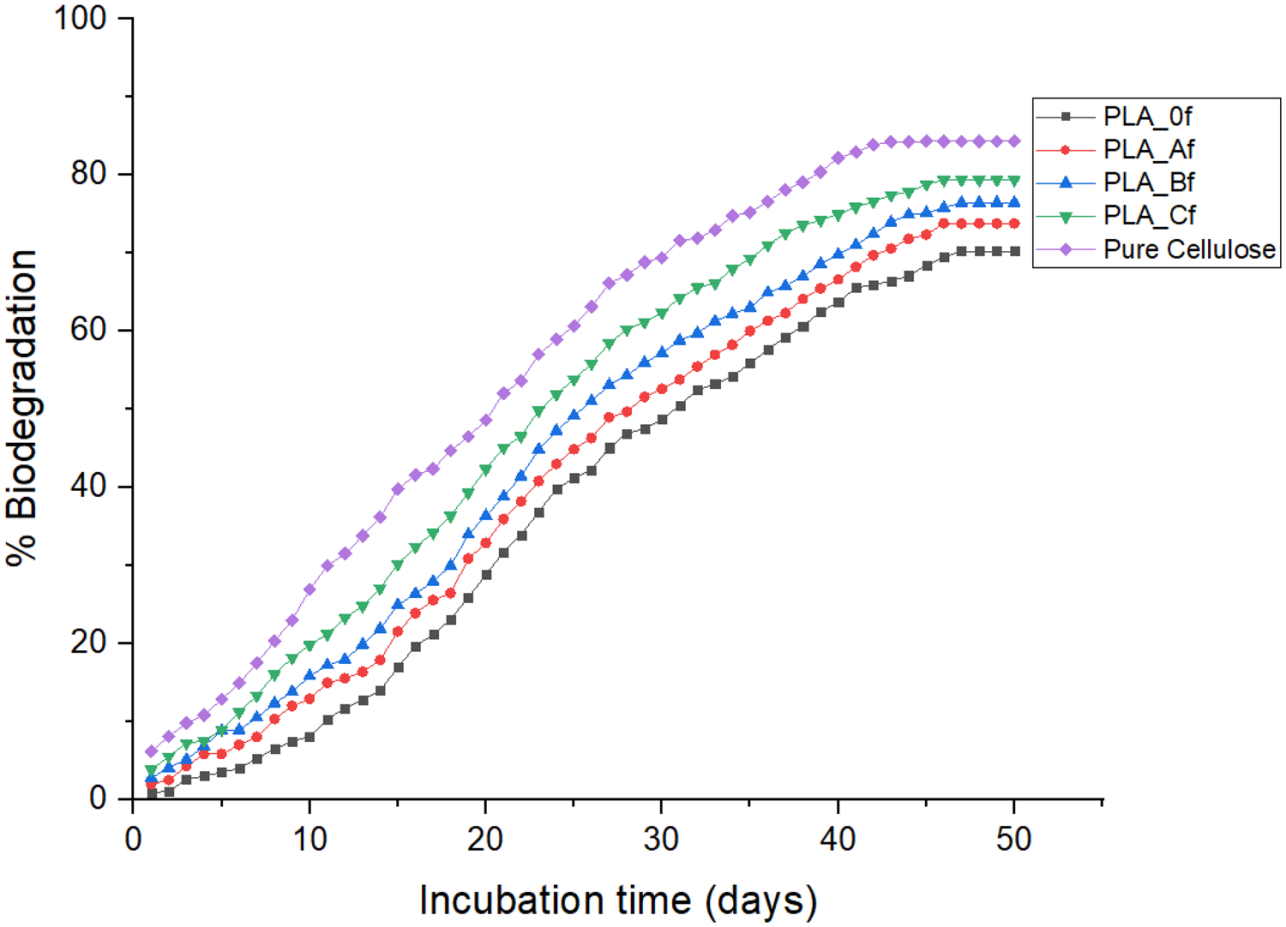
Percentage biodegradation plot for the foams.

Figures 9 and 10 below show the net CO_2_–C produced and percentage biodegradation behavior of the foams over the 50-day period of the composting test. From both figures, it was observed that degradation of PLA_0f. proceeded slowly during the first 8 days compared to PLA_Af before picking up. This suggested that the inclusion of cellulose in PLA foam matrix (although in small concentrations) led to increased microbial activity and facilitated higher mineralization compared to the pure PLA foam. A stabilization in net (CO_2_-C) mg was observed at about the 43rd day (Fig. 9). This showed that although the compost continued to be humidified, maturation phase had been reached and a decline in the biological process was setting in as suggested by the 63% and 70% mineralization observed for both PLA_0f and PLA_Af respectively at this point^146^. At the end of the 50-day incubation period about 70% and 73.2% mineralization was calculated for PLA_0f. and PLA_Af respectively.

Furthermore, PLA_Bf showed a mineralization pattern similar to that of PLA_Af. PLA_Cf with 3 wt. % cellulose content experienced the highest percentage degradation with 79.4% mineralization recorded for it at the end of the composting cycle. This amounted to about 13.4% increase in mineralization compared to PLA_0f^146^. An increase in hydrolytic biodegradation that was facilitated by enzymatic action due to more pathways that were created as the concentration of cellulose fibrils in PLA matrix increased to 3 wt. % was responsible for a corresponding increase in mineralization. For the reference (pure cellulose) about 84% biodegradation that was recorded for it at the end of the experiment suggested that the combined effect of bacterial and esterase enzymes acting on it led to higher degree of hydrolysis and mineralization^147–149^. Table 7 below, which shows the difference in the total organic carbon content (%) of the foams before and after the composting experiment further confirms that an increase in concentration of cellulose concentration in the matrix of PLA enhanced its mineralization.

**Table 7:** Total organic carbon content (%) of the foams.

Sample	Before	After	Reduction%
PLA_0f.	50	31.4	18.6
PLA_Af	49.5	28.6	20.9
PLA_Bf	49.6	17	32.6
PLA_Cf	49.4	13.5	35.9
Pure cellulose	44	14	30

Based on the new ASTM 6400-19 and ISO 17088 international standards which suggest that total carbon in a material or its constituents must have experienced 90% mineralization in a composting test not exceeding 180 days relative to the positive reference for it be referred to as compostable material; compared to the previous standard ASTM 6400-12 which was set at 60% mineralization which is now inactive^146, 150–152^. It can thus be said that a comparative mineralization of about 87%, 91.4% and 95% calculated for PLA_Af, PLA_Bf and PLA_CF when taken as a fractional percentage of the positive reference (cellulose) showed that the inclusion of cellulose fibrils at increased concentrations led the satisfactory compostability of the cellulose reinforced PLA foams during the 50-day experiment. These results have shown that cellulose reinforced PLA foams are compostable and will biodegrade according to international standards when landfilled.

## Conclusion

PLA foams reinforced with micro cellulose fibrils in three different weight percent concentrations (1.5, 2.25 and 3 wt.%) were developed using the solid-state batch foaming process with CO_2_ as the blowing agent. Reduction in the T_g_ values of the foams was an evidence of miscibility and plasticization effect of the fibrils and CO_2_ in amorphous PLA. The micro cellulose fibrils acted as nucleating agents and created numerous nucleation sites by lowering the ${\Delta G}$ value required for bubble nucleation. Higher nucleation efficiency was recorded as weight concentration of MCF increased in PLA causing an increase in cell density of the foams and reduction in cell size when compared to pure PLA foam. Due to the hydrophobic nature of the foaming agent and amorphous PLA, micro cellulose fibrils were loosely held at the interfaces and further perforated the cell walls making it more porous with increased void fraction and reduced cell size and bulk density of the 1.5 wt.% MCF foam (PLA-Af) compared to pure PLA. However, as the concentration of the fibrils in PLA increased, agglomeration at the nodes stiffened the composite by increasing the cell wall thickness which caused an increase in bulk density and foam skin thickness with steady reduction in void fraction and porosity. The increase in percentage open porosity and void fraction recorded for the 1.5 wt.% MCF foam (PLA-Af) alone compared to the other foams was majorly responsible for the improvement in its thermal insulation properties by lowering its thermal conductivity as shown by the theoretical and experimental results. With increasing micro cellulose concentration, thicker skin and cell walls led to a decrease in net void fraction. The increased polymer fraction led to an increase in thermal conductivity over that of the pure PLA foam. Improvement in mechanical properties of the foamed composites was only recorded at low concentration of the fibrils. Poor interfacial bonding and adhesion between the fibrils and PLA matrix in addition to thicker cell walls and the absence of corresponding bigger cell sizes led to reduction in mechanical properties at higher concentrations of the fibrils in the matrix. Slightly higher energy consumption was recorded for the bio-resourced foams over the current polyurethane/ expanded polystyrene insulation with an increase of building heating and cooling loads of about 12%. However, a fully bio-resourced foam with zero VOC manufacturing process would offer environmental benefits with some impact (12%) on energy consumption. The composting results showed that the inclusion of cellulose fibrils in the PLA foam matrix will lead to accelerated biodegradation of the end of the foams when landfilled. Additionally, the results for comparative mineralization of the foams when taken as a fractional percentage of the positive reference also showed that satisfactory compostability according to international standards will occur when they are landfilled at the end of their lives.

## List of symbols

A: Area (cm^2^)
C_wt_: Cell wall thickness (µm)
C_p_: Specific heat capacity at constant pressure (J/kg·K)
Er: Expansion ratio
G: Free Energy (kJ/mol)
M: Magnification (µm)
n: Number of cells
N_f_: Cell Density (cell/µm^3^)
N_s_: Cell Size (µm)
P_foam_: Foaming pressure (MPa)
P_sat_: Saturation pressure (MPa)
R: Thermal resistance value (m·K/W)
SIP: Structural Insulated Panel
sc: Supercritical
t: Sample thickness (m, mm)
T: Temperature (^o^ C)
T_g_: Glass transition temperature (^o^ C)
T_g1_: First cycle T_g_ unfoamed polymer (^o^ C)
T_g2_: Second cycle T_g_ unfoamed polymer (^o^ C)
T_gf1_: First cycle T_g_ foamed polymer (^o^ C)
T_gf2_: Second cycle T_g_ foamed polymer (^o^ C)
T_foam_: Foaming temperature (^o^C)
T_sat_: Saturation temperature (^o^ C)
V_f_: Void fraction (%)
0, A, B, C: Concentration of MCFs in PLA

## Greek symbols

∆(.): Increment of a quantity
ϴ: Volume fraction (%)
ƛ: Thermal Conductivity (W/m·K)
ρ: Density (g/cm^3^)

## Subscript

cv: Convection
f: Foam
g: Gas
p: Polymer
r: Radiation
s: Size
sat: Saturation
sol: Solid phase
1, 2: Indices for cycles
